# Mid-to Long-Term Survival of Total Knee Arthroplasty in Hemophilic Arthropathy

**DOI:** 10.3390/jcm9103247

**Published:** 2020-10-11

**Authors:** Jung-Kwon Bae, Kang-Il Kim, Sang-Hak Lee, Myung-Chul Yoo

**Affiliations:** 1 Department of Orthopaedic Surgery, Kyung Hee University Hospital at Gangdong, Seoul, 05278 Korea; roundnfirm@hanmail.net (J.-K.B.); sangdory@daum.net (S.-H.L.); mcyookuh@naver.com (M.-C.Y.); 2 Department of Orthopaedic Surgery, Kyung Hee University School of Medicine, Seoul 05278, Korea

**Keywords:** hemophilia, knee, arthroplasty, long-term, outcomes, complication, survival

## Abstract

While satisfactory results have been reported during short-to mid-term follow-up assessments of hemophilic patients who have undergone total knee arthroplasty (TKA), limited literature focusing on long-term survival following TKA exists to date. As part of this investigation, a consecutive series of 78 TKAs in 56 patients with hemophilic arthropathy was reviewed. The mean patient age at the time of operation was 38.7 years old and the mean length of follow up was 10.2 years. Clinical and radiologic outcomes, quality of life, complications, and long-term survivorship of TKA were evaluated. At the latest point of follow up, the average American Knee Society (AKS) knee and function scores had improved from 32.1 to 85.7 points and 41.5 to 83.3 points, respectively. Moreover, the average range of motion (ROM) was significantly increased from 64.2° preoperatively to 84.2° postoperatively. The physical and mental Short Form-36 results were also significantly improved at the latest point of follow up. Postoperative complications appeared in 12 knees (15.4%). The readmission rate in the 30 days after discharge was 6.4%. Revision TKA was performed in three knees for periprosthetic joint infection (*n* = 2 knees) and tibial component loosening (*n* = 1 knee). The Kaplan–Meier 10- and 13-year prosthesis survival rates were 97.1% and 93.2%, respectively. The current study suggests that the mid-to long-term results of TKA in patients with hemophilic arthropathy are favorable, with successful long-term prosthesis survival achievable in most cases.

## 1. Introduction

End-stage hemophilic arthropathy of the knee usually leads to total knee arthroplasty (TKA) to reduce severe pain and improve function [1]. However, the execution of TKA in hemophilic arthropathy is complex and challenging due to the anatomical distortion with physeal overgrowth, extensive arthrofibrosis, and poor bone quality [2,3,4]. Furthermore, a distinctive observation exists, contending that TKA in the context of hemophilia is generally performed in young and active male patients due to the nature of the disease [5]. Although several studies with short- to mid-term follow up have demonstrated promising results with high rates of satisfaction together with significant improvement in knee function and pain relief [6,7,8,9], many authors have reported relatively greater rates of early and late complications exist relative to those in nonhemophilic cohorts undergoing TKA, such as aged patients with primary osteoarthritis [10,11,12,13,14]. To date, studies have included only small hemophilic populations with TKA over a long period postoperation at multiple centers and there are little data available reporting long-term results in patients with hemophilia [15,16]. The aim of this study was therefore to evaluate the long-term results of primary TKA in patients with hemophilia performed at a single institution. We also assessed the prosthesis survival rate at longer than 10 years after surgery.

## 2. Materials and Methods

### 2.1. Study Design and Patients

**Figure 1:**
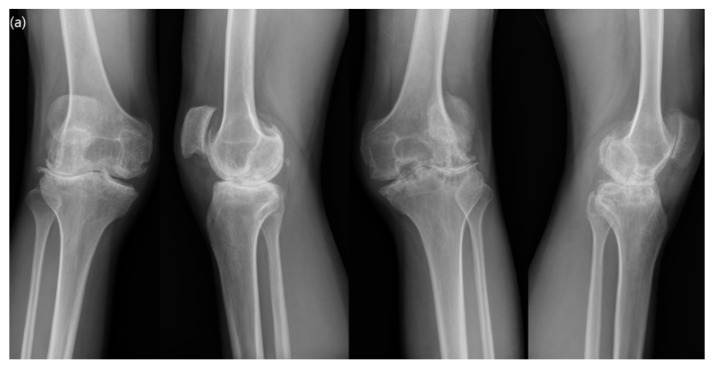

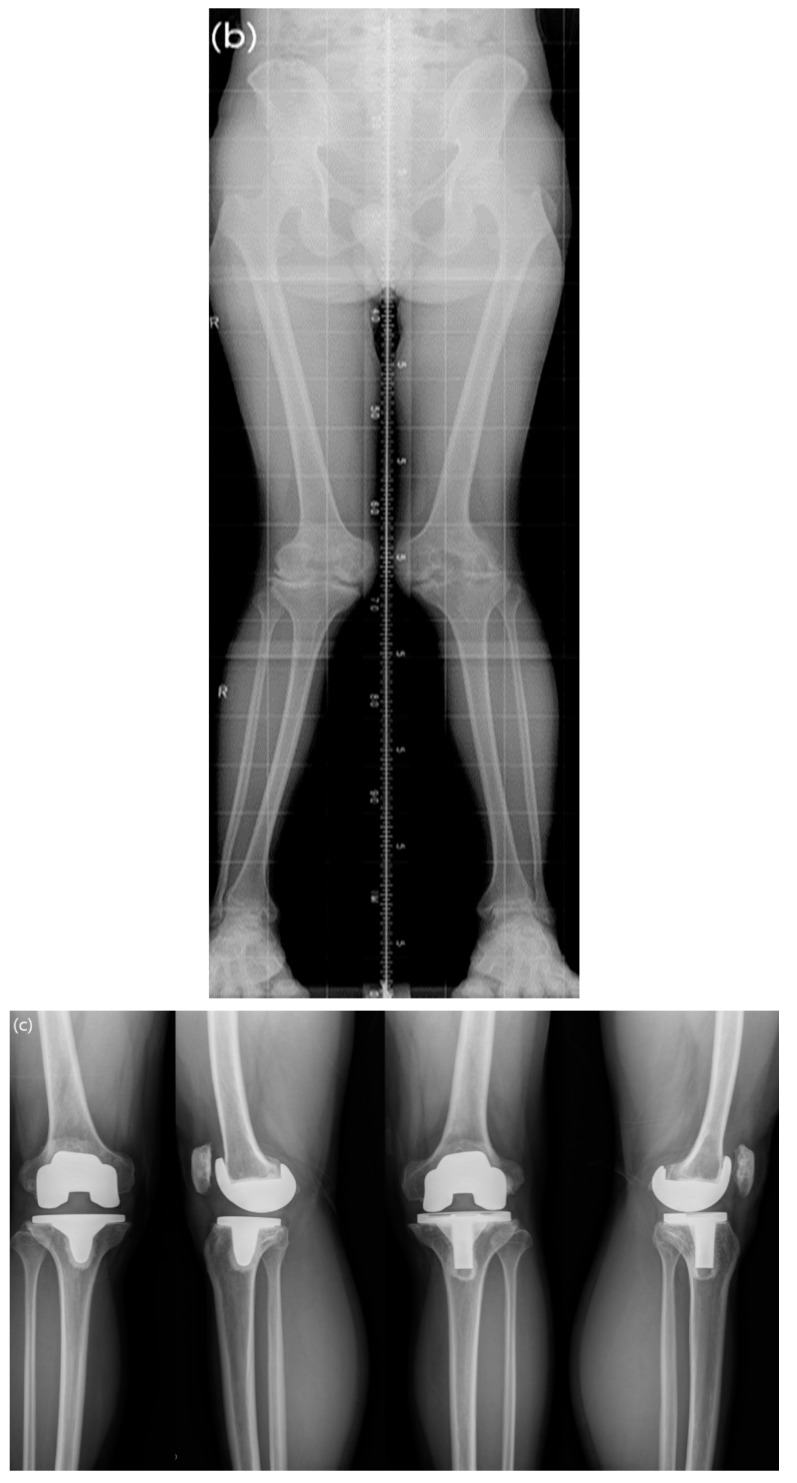

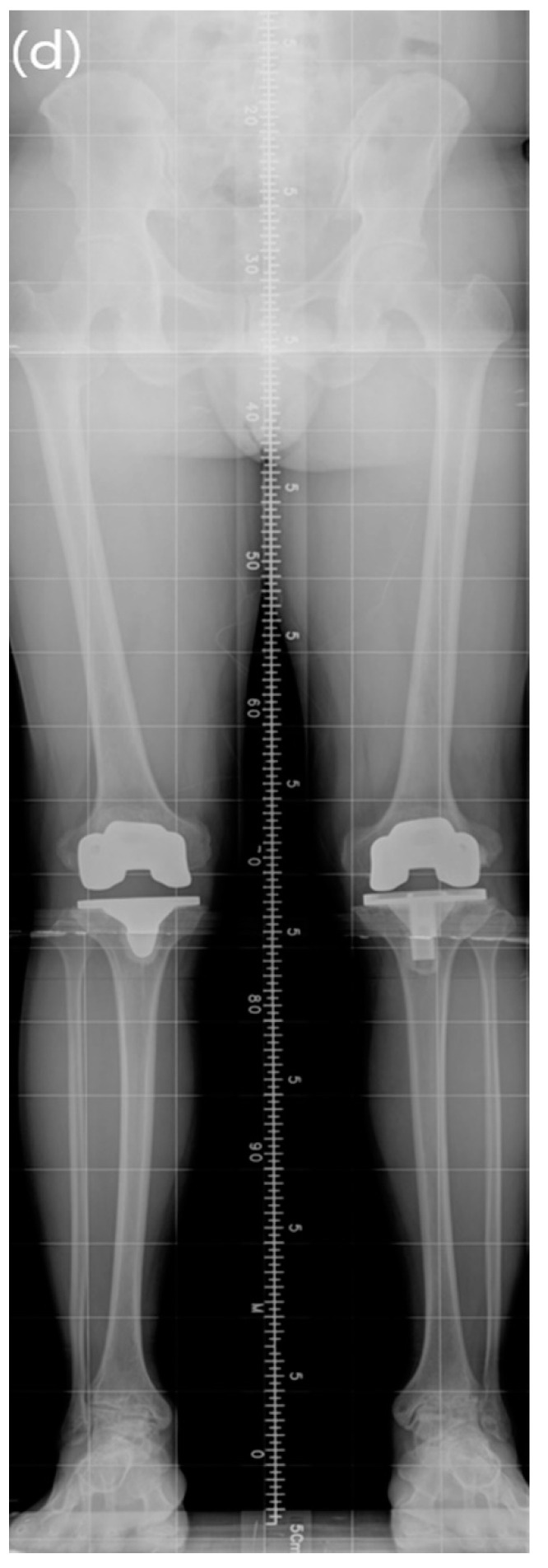
A 36-year-old hemophiliac male with severe valgus deformity. Flexion contractures of both knees were 10° on the right and 15° on the left, respectively. The range of motion (ROM) in both knees was limited to 120°. (**a**) Preoperative standing anteroposterior and lateral radiographs. (**b**) A preoperative lower-extremity orthogram showed 15° valgus of the right lower limb and 14° valgus of the left lower limb. (**c**) A postoperative radiograph taken at 11 years after surgery showed well-positioned components. (**d**) A postoperative lower-extremity orthogram taken at 11 years after surgery revealed neutral alignment in both knees. The ROM of both knees was improved to 140° without flexion contracture.

From February 2007 to May 2015, 102 consecutive TKA procedures were performed in 76 hemophilic patients at a single institute. Indications for TKA were end-stage hemophilic arthropathy with Arnold–Hilgartner stage V [3], functional impairment due to pain, and failure to respond to conservative treatment. All TKA procedures were performed in patients with severe hemophilia (Figure 1). The inclusion criterion for this study was a minimum of five years follow up and, finally, 78 knees in 56 patients were enrolled in the present investigation (Table 1). This study was approved by our institutional review board (KHNMC 2020-07-012).

### 2.2. Operation

All operations were performed by two senior surgeons. Our institution adopts two different computer-assisted surgery (CAS) techniques or conventional surgical techniques when performing TKA in patients with hemophilia. According to surgeon preference, one adopted the computer navigation system (Vector Vision; BrainLAB, Munich, Germany) and the other used a robot-assisted system (ROBODOC, Integrated Surgical Systems, Davis, CA, USA) alternatively. For robot TKA, a single product in each cruciate-retaining (CR) and posterior-stabilized (PS) type was available due to the uploaded system. When CAS techniques were not possible and abandoned during the TKA procedure, we completed the surgery by converting to the conventional technique using the Vanguard knee system (Biomet, Inc., Warsaw, IN, USA). The types of prosthetic components that were used are shown in Table 2.

### 2.3. Hematologic Care

Under the supervision of a specialized, multidisciplinary hemophilia team at our institute, all patients chosen for arthroplasty received factor replacement therapy according to our institute protocol. The factor was administered preoperatively to restore the factor levels to 100% before surgery. Serum levels were maintained at 100% for three days after surgery, at 80% until day 5, at 60% until day 7 after the surgery (Table 3) [17]. Factor VIII was administered three times a day as its half-life is usually eight hours, whereas factor IX was administered twice a day given its half-life is 12 h [18]. Six patients with high titer inhibitor levels were treated with activated recombinant factor VII (rFVIIa) [19].

### 2.4. Postoperative Care

Compressive elastic stockings were applied to all patients to prevent deep vein thrombosis. No other antithrombotic prophylaxis was adopted. The suction drain was usually removed at 48 h postoperatively by considering the bleeding tendency of the surgical wound, and active knee exercise was initiated. At three days postoperatively, the patients were mobilized with crutches. They were typically discharged on day 7 and referred to the clinic at the Korea Hemophilia Foundation for further rehabilitation and factor replacement. The patients participated in sequential follow-up visits at six weeks, three months, six months, and one year after surgery and thereafter were followed up with annually. Clinical and radiographic evaluations were performed at each visit.

### 2.5. Clinical Evaluations and Survival Rate

The American Knee Society (AKS) score was used for clinical evaluation [4], while the Short Form-36 (SF-36) was used for the evaluation of quality of life [20]. According to the AKS function score, an overall result of greater than 85 points was considered as excellent, that of 70 to 84 points was considered as good, that of 60 to 69 points was considered as fair, and that of less than 59 points was considered as poor [21], respectively. Range of motion (ROM) and flexion contracture were measured using a long-armed goniometer. Any postoperative complications and the readmission rate within the 30 days after discharge were recorded. Implant survival at 10 years was also evaluated.

### 2.6. Statistical Analysis

An independent-samples *t*-test was used to compare pre- and postoperative values of AKS, SF-36, ROM, and radiologic parameters. The prosthesis survival rate was evaluated based on the Kaplan–Meier method [22]. The follow-up interval unit was one year and annual success was defined as instances in which the implant remained in place throughout the unit time period. The entire series was included with component removal for infection or any reason acting as the primary endpoints for survivorship analysis. A two-sided *p*-value of less than 0.05 was considered to be statistically significant. All analysis was performed using the Statistical Package for the Social Sciences version 23 software program (IBM Corporation, Armonk, NY, USA).

## 3. Results

### 3.1. Switch to Conventional TKA (Aborting CAS System during TKA)

Twelve cases were switched to conventional technique due to abandoned CAS during the operation. Of these, 10 cases occurred during a robot TKA and two cases occurred during navigation TKA. During robot TKA, six cases were aborted due to registration failure since bony deformities were so severe; the primary designated points for surface registration did not exist. The remaining four cases were aborted due to interruption of the patellar tendon during the milling process. Meanwhile, two cases of navigation TKA were abandoned during the operation, resulted from the registration failure, and converted to conventional technique.

### 3.2. Clinical Outcomes

The mean AKS knee and function score was significantly improved at the latest follow up (Table 4). The overall result was excellent in 65.4% (51/78) and good in 30.8% (24/78) of study participants, respectively. The mental and physical SF-36 scores also increased significantly during follow up (*p* < 0.001). Moreover, the average ROM was significantly increased from 64.2° preoperatively to 84.2° at last follow up (*p* < 0.001). Moreover, six hemophilic patients with inhibitors showed significant clinical improvement similar to the patients without inhibitors compared in Table 4 (Table 5). Despite the existence of severe deformity and bony disruption, lower limb alignment was corrected significantly (*p* < 0.001) following TKA (Table 6).

### 3.3. Complications and Survival Rate

**Figure 2:**
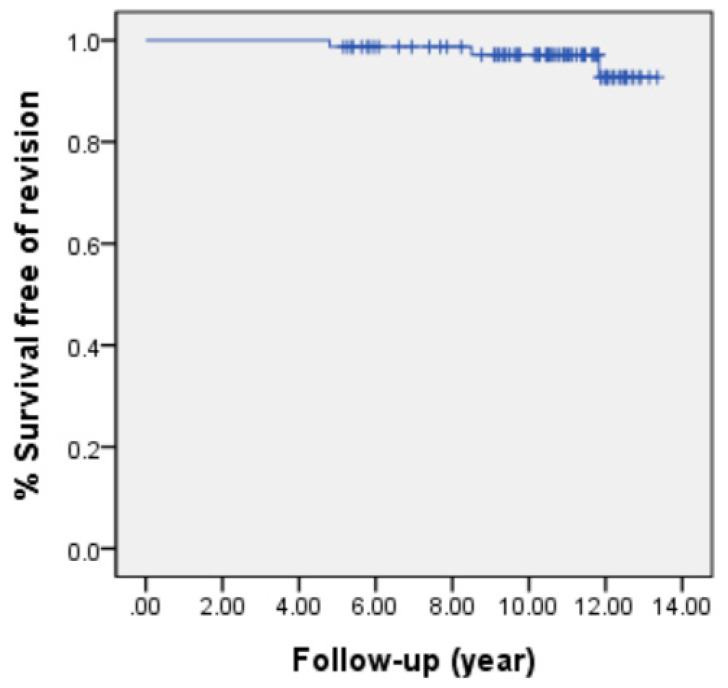
Prosthesis survival after total knee arthroplasty (TKA) in hemophilic arthropathy.

Postoperative complications occurred in 12 knees (15.4%) (Table 7). The readmission rate in the 30 days after TKA was 4.6% (5/78 knees) and the reasons for readmission were hemarthrosis, stiffness, and periprosthetic fracture during rehabilitation. Three knees (3.9%) underwent revision TKA due to periprosthetic joint infection (*n* = 2 knees) or aseptic tibial component loosening at 11.8 years (*n* = 1 knee). At 10 years follow up, 76 prostheses (97.4%) were in place, and 75 prostheses (96.2%) were in place with the 13th year of surveillance. The Kaplan–Meier survivor graph showed 10- and 13-year prosthesis survival rates were 97.1% and 93.2%, respectively (Figure 2).

## 4. Discussion

The main finding of the current study is that mid-to long-term results of TKA in patients with end-stage hemophilic arthropathy were satisfactory in terms of pain relief, functional improvement, and radiologic outcomes. Furthermore, the rate of prosthesis survival by Kaplan–Meier method was excellent as 97.1% and 93.2% at 10 and 13 years postoperatively, respectively.

The long-term survival rate has been well-reported after TKA in nonhemophilic patients [23,24,25] and high rates of aseptic mechanical failure of implants and shorter survival times were reported especially to occur in patients aged 50 years or younger following TKA [26]. Meanwhile, the literature assessing long-term survival following TKAs in patients with hemophilia is limited (Table 8) [15,16,27]. Generally, implant survival in hemophilic patients was considered to be low relative to that in patients without hemophilia because TKA is usually performed at an advanced patient age in individuals with primary osteoarthritis, whereas secondary osteoarthritis due to hemophilia occurs in patients who are relatively young and active male patients [5]. Furthermore, they experienced more difficulty in TKA resulting from extensive arthrofibrosis, severe deformity, and anatomical distortions [2,3,4]. However, despite the above concerns, there have been efforts to improve TKA outcomes in hemophilia patients [28,29] and we were able to obtain successful mid-to long-term outcomes and survivorship. We consider the following reasons to have supported the favorable outcomes in our series. First, to reduce surgical complications in this cohort, we employed CAS techniques in TKA. The use of CAS in TKA may ensure better postoperative alignment of the leg, adequate soft tissue balancing, and proper implant position as compared with TKA performed using a conventional technique and it would be more helpful in cases of severe deformity or limited knee motion [30,31,32,33]. It is also expected that this improved accuracy may lead to better clinical results and reduced revision rates [34,35]. Moreover, since CAS system avoids intramedullary instrumentation, it may decease postoperative bleeding and thromboembolic complication [36,37]. We also observed very low incidence of postoperative hemarthrosis (5.1%) and no cases of symptomatic deep vein thrombosis during the follow-up periods. Even in hemophilic patients, the conduct of TKA using CAS showed promising mid-term outcomes [38,39]. However, twelve cases were switched to conventional technique during the CAS TKA due to several reasons pertaining to an abandoned CAS system such as registration failure or interruption of the patellar tendon during the milling process. It could have happened not only in hemophilia but also in primary osteoarthritis cases [40,41]. Moreover, although the CAS TKA in hemophilia can be useful as demonstrated by current results and the authors could take this into account to improve the outcomes, it is hard to use routinely. Moreover, there has been no clear evidence on superiority of this approach over the conventional technique in patients with hemophilia. Therefore, before using the CAS system, the surgeon should know the benefits and limitations of the CAS system and also should have the ability to perform a switch to conventional TKA when CAS is not feasible during the operation. Further, the favorable results of our series were possible because of the multidisciplinary approach and meticulous attention paid to the surgical technique. Doctors from orthopedic surgery, internal medicine, and the rehabilitation department collaborated together to manage the patient in the perioperative period in terms of coagulation factor level, hematologic assessment, and postoperative rehabilitation. Under the supervision of a specialized, multidisciplinary hemophilia team at our institute, factor replacement was calculated and administered using the standard protocol of our center [17]. The timing of postoperative drain removal and the daily rehabilitation schedule were also adapted to each patient based on our protocol. Meanwhile, patients suffering from hemophilia have learned to adapt to their social environment and life-long challenges [42]. This population tends to have lower subjective expectations and has adjusted to and accepted this [43]. The AKS and SF-36 scores in the present study support the theory that improvement in quality of life after TKA would be the most remarkable aspect and as such seems to have a strong effect on patient satisfaction after TKA [21].

The rate of hemarthrosis after primary TKA in nonhemophilic patients has been reported to range from 0.3% to 1.6% [44,45,46]. However, the reported hemarthrosis rate after TKA in patients with hemophilia is considerably higher than that in nonhemophilic patients [1]. Previous studies have suggested the rate of hemarthrosis, as the most common complication in hemophiliacs, to be 12.5% to 25.6% [13,15,47]. Likewise, postoperative hemarthrosis was the most common complication with an incidence of 5.1% in the present study. This seems to be a slightly higher rate than that reported in nonhemophilic patients but would be comparable with the finding of a previous report on hemophilia [1]. Thus, we think that adopting meticulous surgical techniques with patient-specific rehabilitation and proper factor replacement protocols could reduce the incidence of postoperative hemarthrosis. Meanwhile, the rate of periprosthetic joint infection in patients with hemophilia varies between 1% and 16% [13,14,15,16,21,27,48,49,50] and the rate was 2.6% in the present study. Periprosthetic joint infections in the present study occurred bilaterally in one patient and were diagnosed at postoperative 4.8 and 8.5 years, suggesting the hematogenous spread of infection [21]. Most of these patients were undergoing factor replacement on demand, which might entail a risk for contamination of injections by skin bacteria via the puncture site. Therefore, having an aseptic technique for administering coagulation factor concentrates is important for patients with hemophilia, especially those who have undergone at least one joint replacement [50].

Our study had some limitations. First, the study design was retrospective. Considering the rare incidence of hemophilia, it would be difficult to proceed with such a study as this prospectively in a single institute. Moreover, this was a long-term study and we performed comparisons with previously reported articles offering long-term follow-up data concerning hemophilia. Second, all procedures in our series were performed by two senior surgeons using several implants. Thus, there would be some degree of surgical bias resulting from the involvement of two different surgeons and implants. The presence of different surgeons might be associated with an increased risk of infection or failure [51]. However, as both surgeons in our series are well-experienced arthroplasty surgeons adhering to the same rehabilitation protocols at a single institute, this should not have impaired homogeneity significantly or affected outcomes in the current study. Moreover, even though we used different implants in cases of robotic and navigation TKA according to its program installed, all the implants used have been widely used and proven without differences in the market. Therefore, the bias in the results would be minimal. Third, we could not follow up with all patients through a minimum of five years, resulting in a follow-up rate of 76.5%. Since TKA in hemophilia is a technically demanding procedure and this kind of disease is not as common as primary osteoarthritis in advanced age, patients for this cohort were recruited from all over the country. Ultimately, we reviewed 78 knees in 56 hemophilia patients and this could be considered a large enough number at a single institute. Despite the above limitations, the strength of this study is the successful long-term results and good prosthesis survival rate, which have not been previously reported in a large population at a single institute.

## 5. Conclusions

The current study suggests that mid-to long-term results of TKA in hemophilic arthropathy are favorable with successful long-term prosthesis survival.

## Figures and Tables

**Table 1:** Demographic characteristics.

Patient Details	
Total number	102 knees (76 patients)
Lost to follow up <5 years	20 knees (17 patients)
Deceased within 5 years after TKA	4 knees (3 patients)
Included in the current study	78 knees (56 patients)
Mean follow up (years) ^a^, (range)	10.2 ± 2.22 (5.2–13.4)
Age (years) ^a^, (range)	38.7 ± 8.3 (26–69)
Hemophilia type (A/B)	70 knees/8 knees
Severity (severe/moderate)	75 knees/3 knees
Factor VIII inhibitor positive (%)	6 knees (7.7)
HIV positive (%)	1 knee (1.3)
HCV positive (%)	55 knees (70.5)

^a^ Values are given as mean ± standard deviation.

**Table 2:** The types of prosthetic components used.

Type of Prosthesis		Implant	Number of Knees (PS/CR)
Computer-assisted surgery	Computer-navigated	Triathlon^®^, Stryker PFC^®^, Depuy	14 (17.9)/6 (7.7) 13 (16.7)/1 (1.3)
	Robot-assisted	Nexgen^®^, Zimmer Duracon^®^, Howmedica	19 (24.4)/2 (2.6) 0/11 (14.1)
Conventional		Vanguard^®^, Biomet	12 (15.4)/0-

PS, posterior-stabilized; CR, cruciate-retaining.

**Table 3:** Types of perioperative factor replacement and dosage ^a^.

Type	No. (%) of Knees	Types of Factor Replacement	No. (%) of Knees	Total Amount of Coagulation Factor Concentrates ^b^ (IU/Kg)	
				Operation Day 0–3	Operation Day 4–7
A	70 (89.7)	Greenmono^®^ Monoclate-P^®^ Novoseven^®^^c^ Kogentate^®^	51 (65.4) 11 (14.1) 6 (7.7) 2 (2.6)	375.0 ± 87.4 360.6 ± 93.7 126.0 ± 37.4 445.1 ± 87.1	225.7 ± 59.5 222.8 ± 100.5 103.7 ± 29.6 221.6 ± 4.5 248.1 ± 92.5
B	8 (10.3)	Benefix^®^	8 (10.3)	511.4 ± 131.7	
Total	78 (100)		78 (100)		

^a^ Values are given as numbers. ^b^ Values are given as mean and standard deviation. ^c^ Recombinant factor VIIa, (KIU/Kg) was represented. Greenmono^®^ (Green Cross Corporation, South Korea), Monoclate-P^®^ (CSL Behring, USA), Novoseven^®^ (Novo Nordisk, Denmark), Kogentate^®^ (Bayer, Germany), Benefix^®^ (Bayer, Germany).

**Table 4:** Clinical outcomes between the preoperative and the latest follow up ^a^.

	Preoperative	Last Follow Up	*p*-Value ^b^
Clinical score			
AKS knee score	32.1 ± 5.9	85.7 ± 13.8	<0.001
AKS function score	41.5 ± 9.5	83.3 ± 14.1	<0.001
SF-36 physical score	25.4 ± 14.8	72.2 ± 14.0	<0.001
SF-36 mental score	36.8 ± 20.5	72.9 ± 12.9	<0.001
Functional outcome			
Flexion contracture (°)	19.0 ± 12.4	3.8 ± 6.6	<0.001
Range of motion (°)	64.2 ± 37.9	84.2 ± 32.7	<0.001
The overall result			
Excellent, *n* (%)	–	51 (65.4)	<0.001
Good, *n* (%)	–	24 (30.8)	<0.001
Fair, *n* (%)	18 (23.1)	2 (2.6)	<0.001
poor, *n* (%)	60 (76.9)	1 (1.3)	<0.001

AKS, American Knee Society knee score; SF-36, Short Form-36 score. ^a^ Values are given as mean ± standard deviation. ^b^ The Student’s *t*-test was used to compare continuous variable outcomes between groups.

**Table 5:** Clinical outcomes between the preoperative and the latest follow up in patients with inhibitors ^a^.

	Preoperative	Last Follow Up	*p*-Value ^b^
Clinical score			
AKS knee score	30.7 ± 7.3	82.0 ± 21.6	<0.001
AKS function score	40.5 ± 8.8	81.2 ± 22.0	<0.001
SF-36 physical score	24.4 ± 7.6	71.9 ± 9.1	<0.001
SF-36 mental score	36.2 ± 14.4	72.5 ± 11.7	<0.001
Functional outcome			
Flexion contracture (°)	19.2 ± 10.7	4.3 ± 4.2	<0.001
Range of motion (°)	56.7 ± 31.4	73.3 ± 37.9	<0.001
The overall result			
Excellent, *n* (%)	–	4 (66.6)	<0.001
Good, *n* (%)	–	2 (33.3)	<0.001
Fair, *n* (%)	–	–	<0.001
poor, *n* (%)	6 (100)	–	<0.001

AKS, American Knee Society knee score; SF-36, Short Form-36 score. ^a^ Values are given as mean and standard deviation. ^b^ Student’s *t*-test was used to compare continuous variable outcomes between groups.

**Table 6:** Radiologic results for lower limb alignment ^a^.

	Preoperative	Last Follow Up	*p*-Value ^b^
Lower limb alignment			
Varus knee (*n* = 33) (°)	−6.92 ± 3.71 (−0.7 to −16.6)	−0.90 ± 2.73 (−5.3 to 4.77)	<0.001
Valgus knee (*n* = 45) (°)	6.57 ± 5.20 (0.3 to 19.3)	0.62 ± 2.47 (−2.52 to 4.35)	<0.001

^a^ Values are given as mean ± standard deviation. ^b^ The Student’s *t*-test was used to compare continuous variable outcomes between groups. Values are given as mean ± standard deviation; “+” indicates a valgus angle and “−“ indicates a varus angle.

**Table 7:** Postoperative complication after primary total knee arthroplasty (TKA) in hemophilic arthropathy ^a^.

Complication	Number of Knees (%)	Management
Hemarthrosis	4 (5.1)	Arthroscopic lavage Incision and drainage Extra dosing of coagulation factors
Stiffness	2 (2.6)	Manipulation under anesthesia
Periprosthetic fracture	2 (2.6)	Internal fixation
Wound dehiscence	1 (1.3)	Secondary closure
PJI	2 (2.6)	Two-stage revision TKA
Implant loosening	1 (1.3)	Revision TKA

PJI, periprosthetic joint infection. ^a^ Values are given as numbers.

**Table 8:** Comparison of the long-term results of previously-reported TKA in hemophilic arthropathy ^a.^

Study	Number of Knees	Mean Follow Up (Years)	Infection Rate (%)	Loosening Rate (%)	10-Year Survival Rate (%)
Wang et al. [15] (2012)	40	11.8	12.5	2.5	83
Westberg et al. [16] (2014)	107	11.2	6.5	13.1	88
Santos Silva [27] (2019)	18	11.3	11.2	0	94.3
Current study	78	10.2	2.6	1.3	97

^a^ Values are given as numbers.
